# CD40- and 41BB-specific antibody fusion proteins with PDL1 blockade-restricted agonism

**DOI:** 10.7150/thno.66119

**Published:** 2022-01-01

**Authors:** Juliane Medler, Kirstin Kucka, Vinicio Melo, Tengyu Zhang, Stefan von Rotenhan, Jakob Ulrich, Edwin Bremer, Michael Hudecek, Andreas Beilhack, Harald Wajant

**Affiliations:** 1 Division of Molecular Internal Medicine, Department of Internal Medicine II, University Hospital Würzburg, Würzburg, Germany.; 2 Department of Hematology, University of Groningen, University Medical Center Groningen, Groningen, The Netherlands.; 3 Medizinische Klinik und Poliklinik II, Universitätsklinikum Würzburg, Würzburg, Germany.; 4 Interdisciplinary Center for Clinical Research Laboratory, Department of Internal Medicine II, University Hospital Würzburg, Würzburg, Germany.

**Keywords:** 41BB, bispecific antibody, CD40, PDL1, TNFRSF

## Abstract

**Background:** A strategy to broaden the applicability of checkpoint inhibitors is the combined use with antibodies targeting the immune stimulatory receptors CD40 and 41BB. However, the use of anti-CD40 and anti-41BB antibodies as agonists is problematic in two ways. First, anti-CD40 and anti-41BB antibodies need plasma membrane-associated presentation by FcγR binding to exert robust agonism but this obviously limits their immune stimulatory efficacy by triggering ADCC, CDC or anti-inflammatory FcγRIIb activities. Second, off tumor activation of CD40 and 41BB may cause dose limiting systemic inflammation.

**Methods:** To overcome the FcγR-dependency of anti-41BB and anti-CD40 antibodies, we genetically fused such antibodies with a PDL1-specific blocking scFv as anchoring domain to enable FcγR-independent plasma membrane-associated presentation of anti-CD40- and anti-41BB antibodies. By help of GpL-tagged variants of the resulting bispecific antibodies, binding to their molecular targets was evaluated by help of cellular binding studies. Membrane PDL1-restricted engagement of CD40 and 41BB but also inhibition of PDL1-induced PD1 activation were evaluated in coculture assays with PDL1-expressing tumor cell lines and 41BB, CD40 and PD1 responsible cell lines or T-cells.

**Results:** The binding properties of the bispecific antibody fusion proteins remained largely unchanged compared to their parental molecules. Upon anchoring to membrane PDL1, the bispecific antibody fusion proteins activated CD40/41BB signaling as efficient as the parental anti-CD40/anti-41BB antibodies when bound to FcγRs or cells expressing membrane-bound CD40L/41BBL. PD1 inhibition remained intact and the anti-41BB fusion protein thus showed PDL1-restricted costimulation of T-cells activated in vitro with anti-CD3 or a BiTe.

**Conclusions:** Targeting of anti-CD40 and anti-41BB fusion proteins to membrane PDL1 with a blocking PDL1 scFv links PD1-PDL1 checkpoint blockade intrinsically with engagement of CD40 or 41BB.

## Introduction

Activation of the immune checkpoint receptors cytotoxic lymphocyte-associated antigen 4 (CTLA-4) and programmed cell death protein 1 (PD1) on T-cells can crucially promote tumor immune evasion. Indeed, the approval of blocking antibodies against CTLA-4, PD1 or its ligand PDL1 was a major breakthrough in immune therapy and had a strong impact on the treatment of patients suffering on metastatic melanoma, lung cancer and several other tumor entities 1-3. Despite the impressive clinical efficacy, which can be achieved with checkpoint inhibitors, many patients fail to respond satisfactorily and display lack of efficacy and/or side effects 1,4,5. A major factor causing checkpoint inhibitor resistance is presumably the lack of an intratumoral antitumor response due to the presence of immunosuppressive cells such as myeloid derived suppressor cells or regulatory T-cells. Cotreatment regimes with immune stimulatory reagents, e.g. agonistic antibodies targeting CD40 and 41BB, are therefore considered as possible options to broaden the clinical applicability of checkpoint inhibitors 4,5. Indeed, in various mouse cancer models combined treatment with αPD1, αPDL1 or αCTLA4 antibodies along with CD40- or 41BB-specific antibodies resulted in enhanced antitumor activity 4-7.

Agonistic antibodies targeting CD40 or 41BB have been intensively studied preclinically but also in clinical trials 6-9. Despite the observation of profound anti-tumoral effects in animal models, the clinical trials showed no or only modest therapeutic effects and in some cases dose limiting toxicity. Thus, there is no approved use of this type of reagents in the clinic. The lack of success of agonistic anti-CD40 and anti-41BB antibodies to date has two main causes: 1. CD40 and 41BB belong to a category of receptors of the TNF (tumor necrosis factor) receptor superfamily (TNFRSF) which typically become only be efficiently stimulated by IgG antibodies when these antibodies are presented as membrane-bound molecules due to their interaction with Fcγ receptors 10. The dependency of the agonism of αCD40 and α41BB antibodies from the presentation by Fcγ receptors now causes various limitations. Both the presence of immune cells expressing FcγRs and the FcγR expression levels of these cells obviously place an upper limit for the achievable agonistically active antibody species and thus act as a bottleneck for agonistic activity. The “agonistic” presentation of αCD40 and α41BB IgG antibodies by FcγRs is furthermore inevitably connected with the triggering of FcγR signaling, which in turn can result in activities counteracting the desired therapeutic effects. 2. Systemic activation in particular of CD40 might trigger dose-limiting side effects outside the tumor 11.

The FcγR-dependency of the agonism of αCD40 and α41BB antibodies is neither restricted to a certain type of FcγR nor requires engagement of FcγR-associated signaling pathways. This suggests that it is the sheer presentation in cell-bound form that is responsible for the high agonistic activity of FcγR-bound αCD40- and α41BB antibodies. In accordance with this idea, we recently found that antibody fusion proteins harboring an anchoring domain (AD), which recognizes a cell surface-exposed anchoring target (AT), display strong AT-dependent agonism resembling those of the FcγR-bound antibodies 12,13. The much higher agonistic activity of FcγR- and AT-bound antibodies and antibody fusion proteins correspond well with the fact that the natural activators of CD40 and 41BB, the ligands CD40L and 41BBL, are also much more active as membrane-bound molecules than in soluble form.

In this study, we aimed on to exploit the high agonism of AT-bound αCD40- and α41BB-AD fusion proteins to generate CD40 and 41BB agonists that not only intimately link checkpoint inhibition with CD40/41BB engagement but also act preferentially in the tumor to reduce so potential off-tumor effects. For this purpose, we generated CD40- and 41BB-specific antibody variants with an anchoring domain (AD) which binds to the cell surface-exposed checkpoint molecule PDL1 which is preferentially expressed in the tumor. Either a PDL1 recognizing scFv of the PDL1-specific antibody Avelumab was fused as an AD to αCD40/41BB antibodies or CD40/41BB-specific scFvs were fused to the C-terminus of αPDL1. All molecules obtained acquired high FcγR-independent CD40/41BB-stimulating activity upon binding to PDL1. This not only confirmed the crucial role of cell surface presentation of anti-CD40/41BB antibodies for exerting agonistic activity but also offer straightforwardly a concept to avoid the negative effects related to the FcγR-dependency of the agonism of conventional αCD40/41BB antibodies.

## Results

### Bispecific antibody variants targeting CD40 and PDL1 display strong PDL1-restricted CD40 agonism

**Figure 1:**
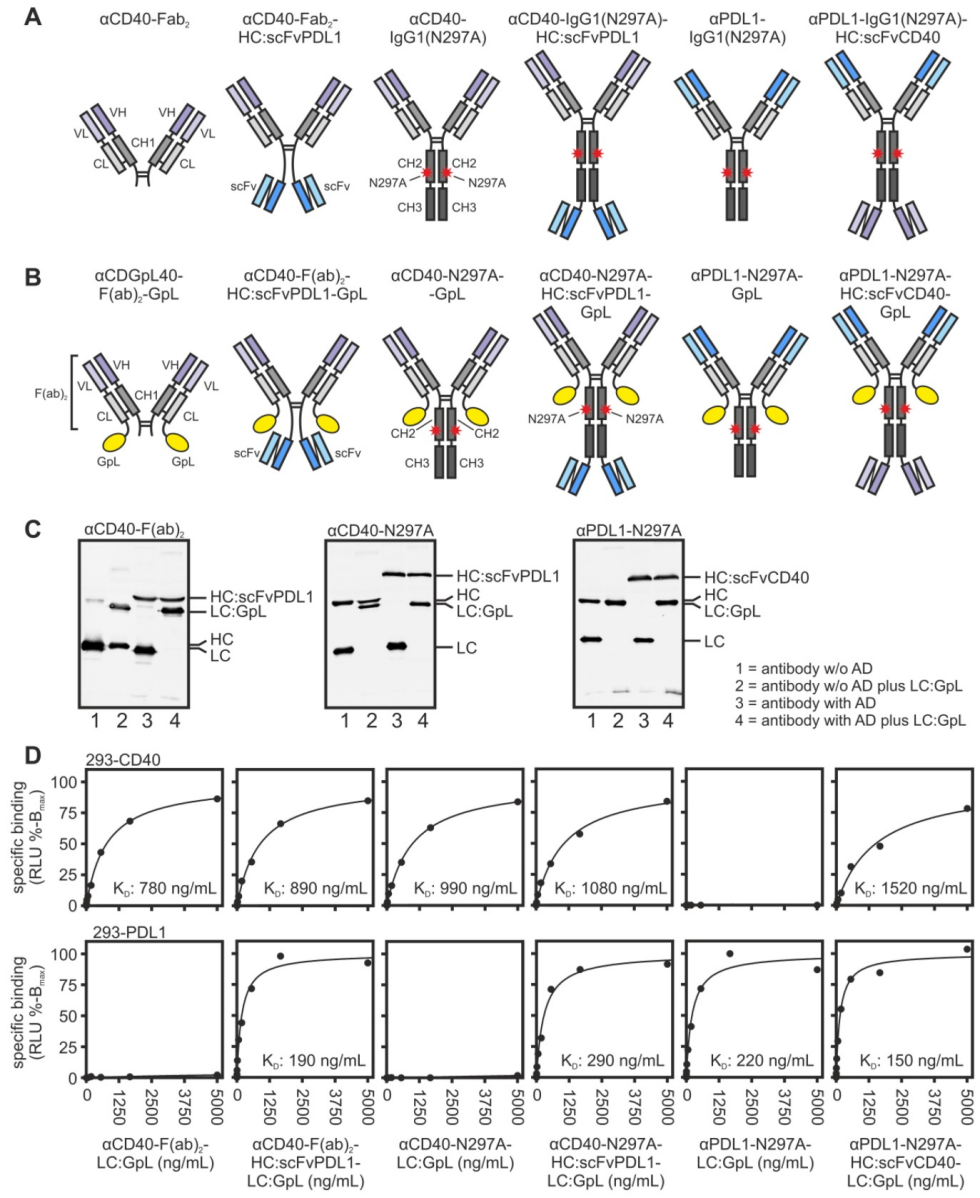
** Equilibrium binding of CD40/PDL1-bispecific antibody variants and the corresponding parental antibodies to their cell-expressed target molecules. (A,B)** Domain architecture of the various antibodies variant (A) and the corresponding GpL fusion proteins (B). N297A indicates a point mutation which prevents or strongly reduces binding to FcγRs. Heavy and light chains of all constructs contained an N-terminal Flag tag for estimation of concentration in cell culture supernatants and gentle purification. CH1/2/3, heavy chain constant region 1/2/3; CL, light chain constant region; Fab, antigen binding fragment; scFv, single chain variable fragment; VH, heavy chain variable region; VL light chain variable region. **(C)** 200 ng of the indicated antibody variants were separated by SDS-PAGE and visualized by western blotting with αFlag. **(D)** Specific binding of the GpL-tagged antibody variants to CD40- and PDL1-expressing cells. One representative experiment is shown. Mean and single K_D_-values of three to six independent experiments are summarized in Table 1 of the manuscript.

To obtain CD40/PDL1-bispecific antibodies variants devoid of the ability to interact with FcγRs, we fused a single chain variable fragment (scFv)-domain derived of the anti-PDL1 antibody Avelumab to the C-terminus of the heavy chain of the Fab_2_ domain or the IgG1(N297A) variant of the anti-CD40 antibody C 14 (Figure 1A). Complementary to the later, a scFv-domain derived of the anti-CD40 antibody C was C-terminally fused to anti-PDL1-IgG1(N297A) (Figure 1A). The resulting antibody fusion proteins αCD40-Fab_2_-HC:scFvPDL1, αCD40-IgG1(N297A)-HC:scFvPDL1 and αPDL1-IgG1(N297A)-HC:scFvCD40 were initially compared with their parental molecules αCD40-Fab_2_, αCD40-IgG1(N297A) and αPDL1-IgG1(N297A) with respect to binding to CD40 and PDL1.

For this purpose, all antibody variants were produced with genetically modified light chains harboring a C-terminal *Gaussia princeps* luciferase (GpL) reporter domain for simple quantification (Figure 1B and 1C). Cellular binding studies with HEK293 cells transiently transfected with CD40- and PDL1 expression plasmids showed that the αCD40-Fab_2_ domain in all constructs and also the two scFv:CD40 domains present in αPDL1-IgG1(N297A)-HC:scFvCD40 have a comparable affinity for CD40 in the range of app. 1000 ng/mL (Figure 1D and S1, Table 1). Likewise, all antibody variants containing the αPDL1-Fab_2_ domain or two scFv:PDL1 domains showed high affinity binding to PDL1 with a K_D_-value of app. 200 ng/ml (Figure 1D and S1, Table 1). This indicates that the Fab_2_ and scFv domains combined in the various bispecific antibody constructs maintained the binding properties of their parental antibody molecules.

**Figure 2:**
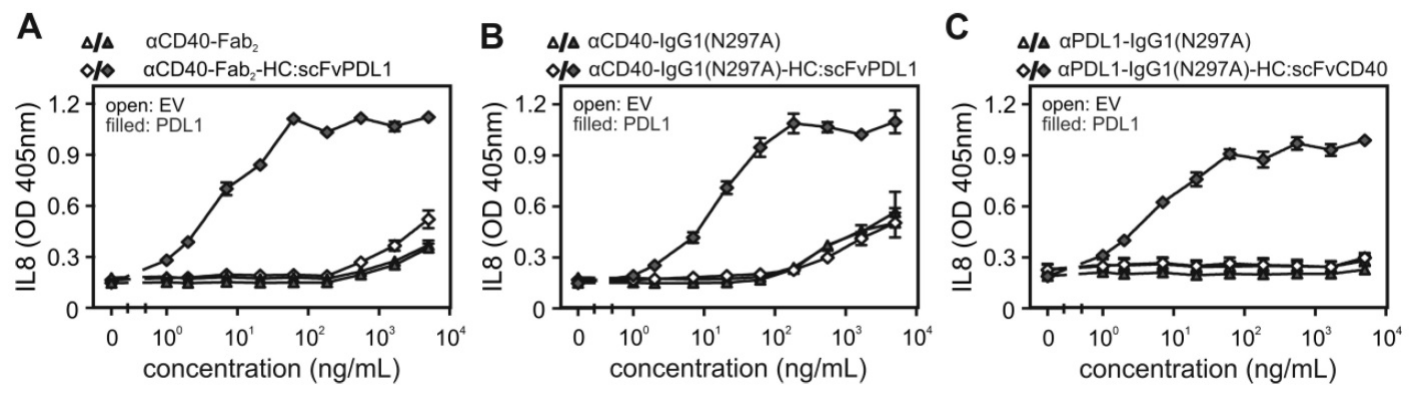
** PDL1-dependent CD40 agonism of CD40/PDL1-bispecific antibody variants. (A, B, C)** HT1080-CD40 cells were stimulated in the presence of HEK293 cells transfected with empty vector (EV) or an PDL1-encoding expression plasmid with the indicated concentrations of αCD40-Fab_2_-HC:scFvPDL1 and αCD40-Fab_2_ (**A**), αCD40-IgG1(N297A)-HC:scFvPDL1 and αCD40-IgG1(N297A) (**B**) or αPDL1-IgG1(N297A)-HC:scFvCD40 and αPDL1-IgG1(N297A) (**C**). Next day, CD40 activation was quantified by measuring upregulation of IL8 production by ELISA. Shown are data obtained by three independent experiments.

Next, we investigated whether the binding of the CD40/PDL1-bispecific antibody variants to cell expressed PDL1 has an effect on the ability of these molecules to engage CD40 signaling. Cell culture supernatants containing the various antibody variants were added to HT1080 cells stably expressing CD40 (HT1080-CD40; 15) together with HEK293 cells transiently transfected with a PDL1 expression plasmid or empty vector (EV). Upon stimulation of CD40 HT1080-CD40 cells produce high amounts of IL8 by far exceeding the IL8 production of HEK293 cells 12,15. IL8 was therefore assayed the next day and were used to quantify CD40 activation. These experiments showed a clear and coherent result. All CD40/PDL1-bispecific antibody variants triggered strong IL8 production with low EC-values of 10-20 ng/mL in the presence of PDL1-expressing HEK293 cells (Figure 2A-C). In contrast, in the presence of EV-transfected HEK293 cells half maximal IL8 induction was even not reached at > 100 fold higher concentrations (Figure 2A-C). Indeed, in the absence of PDL1-expressing cells, the CD40/PDL1-bispecific antibody variants were as poorly active as αCD40-Fab_2_ (Figure 2A) and αCD40-IgG1(N297A) (Figure 2B).

**Figure 3:**
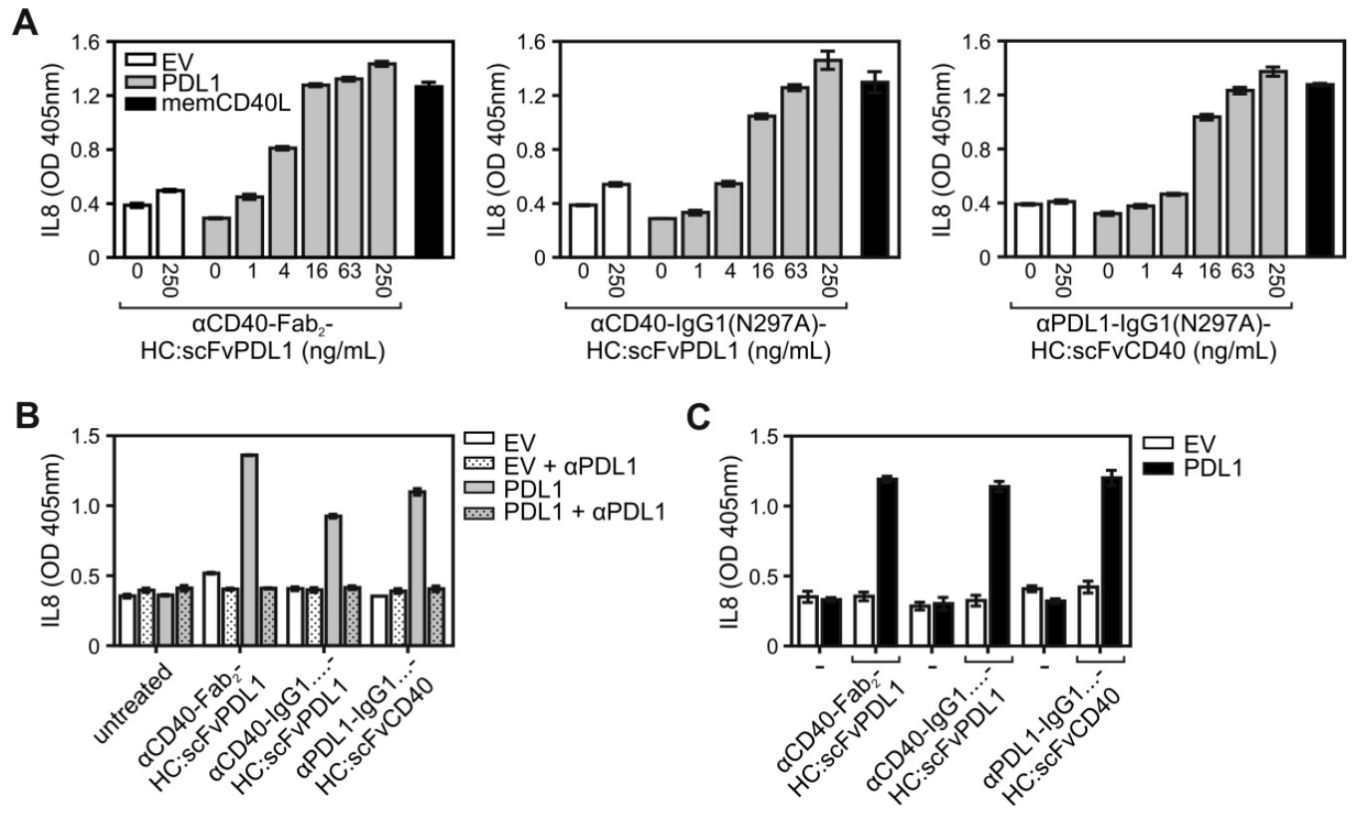
** CD40 activation by membrane CD40L-expressing cells and PDL1-anchored CD40/PDL1-bispecific antibody variants. (A)** HT1080-CD40 cells were stimulated with empty vector- (open bars) and PDL1-transfected (grey bars) HEK293 cells along with the indicated concentrations of the various antibody fusion proteins or with HEK293 cells transfected with a membrane CD40L expression vector (black bars). After overnight incubation IL8 was measured by ELISA. **(B)** Empty vector- (EV) and PDL1-transfected HEK293 cells were pre-incubated with 40 µg/mL of the parental anti-PDL1 antibody (αPDL1) and were then added together with the indicated CD40-targeting antibody fusion protein (10 ng/mL) to HT1080-CD40 cells. Next day, IL8 production was again determined as a readout of CD40 activity. **(C)** U2OS cells expressing endogenous CD40 were stimulated with empty vector- (open bars) and PDL1-transfected (black bars) HEK293 cells along with 200 ng/mL of the different antibody fusion proteins and next day IL8 production was evaluated. Experiments shown in A to C are representative for two to three independent experiments.

Naturally, signaling by CD40 is engaged by the membrane-bound form of its ligand CD40L (memCD40L). To compare the CD40-stimulatory activity of PDL1-anchored αCD40 antibody fusion proteins with those of memCD40L, HT1080-CD40 cells were cocultured on the one hand with EV- or PDL1-transfected HEK293 cells in the presence of increasing concentrations of the various CD40/PDL1-bispecific antibody variants or on the other hand with memCD40L-transfected HEK293 cells. Noteworthy, the maximal IL8 responses induced by the PDL1-anchored CD40/PDL1-bispecific antibody variants were comparable to the IL8 response induced by memCD40L expressing HEK293 cells (Figure 3A). In accordance with the PDL1-dependency of the CD40 agonism of the CD40/PDL1-bispecific constructs, preincubation with the parental PDL1-specific antibody completely abrogated the CD40-mediated IL8 responses of the CD40/PDL1-bispecific antibody variants in HT1080-CD40 (Figure 3B). To investigate the PDL1-restricted agonsim of CD40/PDL1-bispecific antibody variants on cells endogenously expressing CD40, coculture assays with EV- or PDL1-transfected HEK293 cells were additionally performed with U2OS cells (Figure 3C). The results also indicated a strong PDL1-restricted CD40 agonsim.

### Bispecific antibody variants targeting 41BB and PDL1 display strong PDL1-restricted 41BB agonism

The receptors of the TNFRSF (TNFRs) can be classified into two categories based on their ability to become activated by soluble ligand trimers and bivalent antibodies. A first category of TNFRs is already efficiently activated by binding soluble ligand trimers and bivalent antibodies, whereas the receptors of a second category of TNFRs are only strongly stimulated by such reagents when they are presented in oligomerized or plasma membrane-bound form 10,12. In addition to CD40, this second category of TNFRs also includes other important immunoregulatory receptors such as CD27, OX40, TNFR2 and 41BB 10,12. We therefore evaluated the possible PDL1-dependent TNFR agonism of 41BB/PDL1-bispecific antibody proteins in a similar fashion as before for the CD40/PDL1-bispecific antibody variants. Cellular binding studies with HEK293 cells transiently expressing 41BB- and PDL1 again revealed high affinity binding of GpL fusion proteins of the various bispecific constructs to their targets (Figure S2 and S3, Table 2). While there was no significant difference in the affinity of the 41BB binding sites for 41BB between the parental antibody (α41BB-IgG1(N297A) and the two bispecific IgG1(N297A) variants (α41BB-IgG1(N297A)-HC:scFvPDL1; αPDL1IgG1(N297A)-HC:scFv41BB), the binding of the α41BB-Fab_2_ variants was moderately but significantly reduced (Figure S3). Nevertheless, despite the minor differences in the affinity of their 41BB binding domain to 41BB, this set of bispecific antibody constructs largely maintained their binding abilities, too.

**Figure 4:**
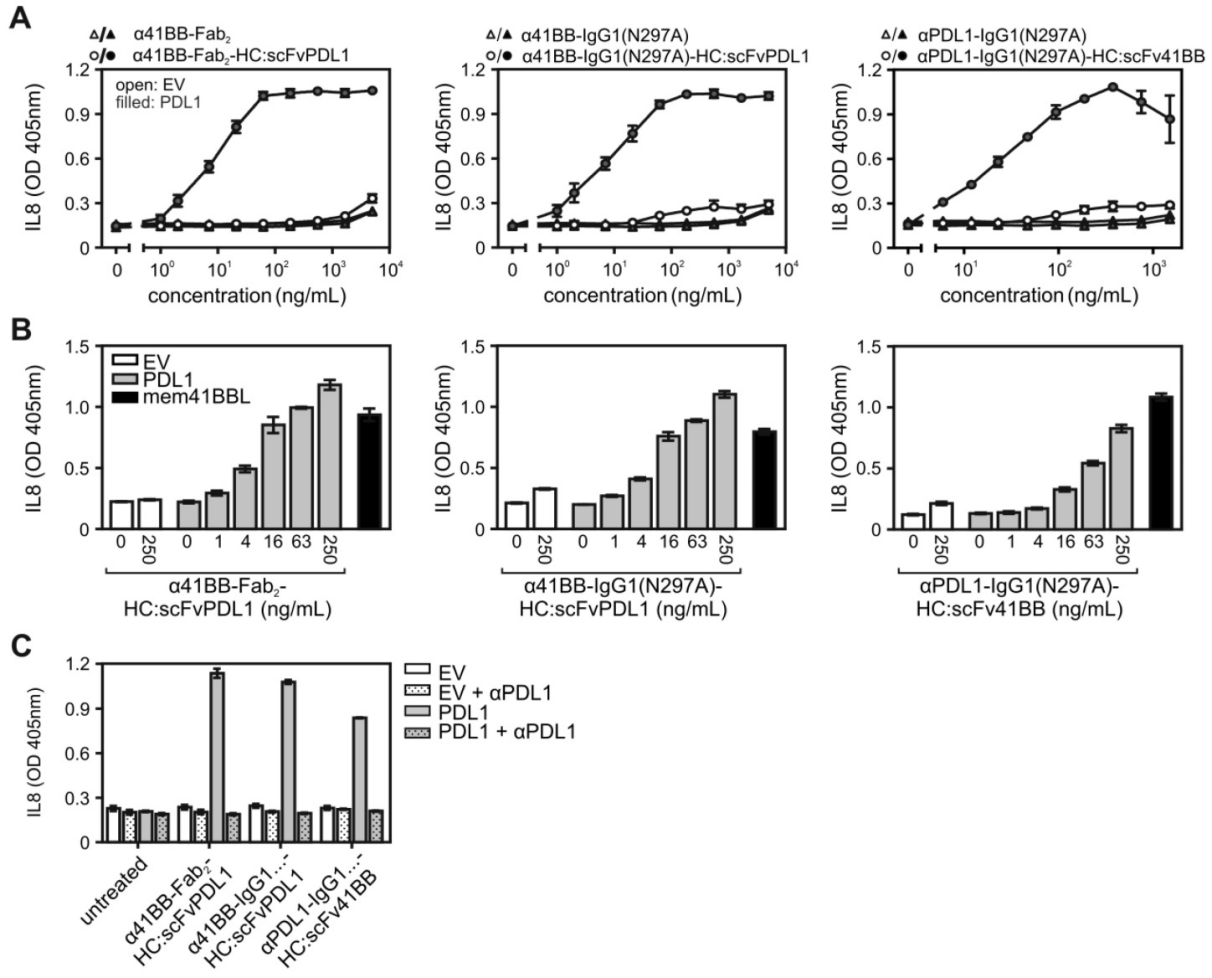
** PDL1-dependent 41BB agonism of 41BB/PDL1-bispecific antibody variants. (A)** HT1080-41BB cells were stimulated in the presence of EV- and PDL1-transfected HEK293 cells with increasing concentrations of the indicated antibody variants. Next day, 41BB activation was evaluated by determination of IL8 production. (**B**) HT1080-41BB cells were stimulated with empty vector- (open bars) and PDL1-transfected (grey bars) HEK293 cells along with the indicated concentrations of α41BB-Fab_2_-HC:scFvPDL1, α41BB-IgG1(N297A)-HC:scFvPDL1 and αPDL1-IgG1(N297A)-HC:scFv41BB or with HEK293 cells which have been transfected with a membrane 41BBL expression vector (black bars). Next day, IL8 was measured by ELISA. **(C)** EV- and PDL1 transfected HEK293 cells were pre-incubated with 40 µg/mL of the parental PDL1-specific antibody (αPDL1) and were then transferred along with the antibody and 10 ng/mL of the 41BB/PDL1-bispecific constructs to HT1080-41BB cells. Next day, IL8 production was measured*.* For (A) three independent experiments have been averaged, for (B) and (C) one representative experiment of two with technical triplicates is shown.

Stimulation of cocultures of HT1080 cells stably transfected with 41BB (HT1080-41BB; 15) with HEK293 cells transiently transfected with EV or a PDL1 expression plasmid revealed efficient PDL1-dependent engagement of 41BB by all three 41BB/PDL1-bispecific antibody variants (Figure 4A). Worth mentioning, the PDL1-anchored 41BB/PDL1-bispecific antibody variants were again as potent as the membrane-expressed 41BBL molecule in triggering 41BB-mediated IL8 production (Figure 4B) and preincubation with the parental PDL1-specific antibody completely inhibited the IL8 responses of the 41BB/PDL1-bispecific antibody variants (Figure 4C).

### Purification and bifunctionality of purified CD40/PDL1- and 41BB/PDL1-bispecific antibody fusion proteins

**Figure 5:**
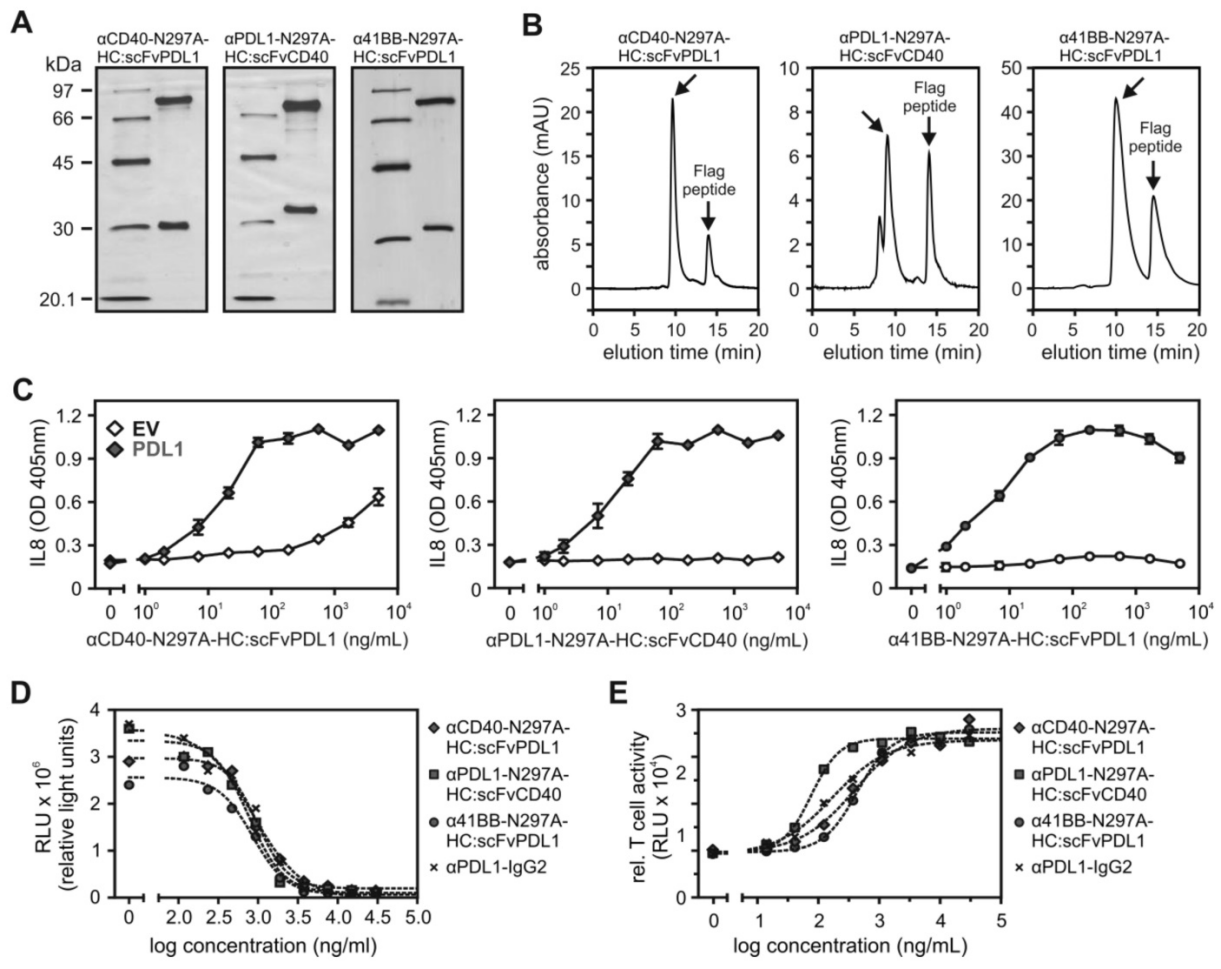
** CD40/PDL1- and 41BB/PDL1-bispecific antibody fusion proteins block PDL1-PD interaction. (A)** Affinity purified proteins (200 ng) were separated by SDS-PAGE and visualized by silver staining: **(B)** Gel filtration analysis of the indicated purified antibody fusion proteins. Arrows indicate peaks of non-aggregated protein species. Also remaining Flag peptide from the affinity purification is marked. **(C)** PDL1-dependent TNFR agonism of the purified proteins were evaluated by coculture assays of CD40- or 41BB-expressing HT1080 variants with empty vector (EV)- and PDL1-transfected HEK293 cells. After overnight stimulation TNFR activation was measured by IL8 ELISA. (**D**) Binding of PD1-GpL (300 ng/mL) to PDL1-expressing HEK293 transfectants was determined in the presence and absence of the indicated antibody variants. **(E)** TCR^+^PD1^+^ Jurkat cells expressing luciferase under the control of a NFAT response element were co-cultivated with CHO-K1 cells expressing a TCR agonist and PDL1. Cocultures were treated with the indicated concentrations of different PDL1-targeting antibody fusion proteins for 6 hours and NFAT-driven luciferase expression was measured. For (**C**) three independent experiments have been averaged, for (**D, E**) one representative experiment of three is shown.

The experiments presented to this point were carried out with supernatants containing the bispecific antibody fusion proteins of interest. To verify that purified antibody fusion proteins maintain integrity and do not aggregate, we exemplarily purified αCD40-IgG1(N297A)-HC:scFvPDL1, αPDL1-IgG1(N297A)-HC:scFvCD40 and α41BB-IgG1(N297A)-HC:scFvPDL1 by gravity flow affinity chromatography on αFlag antibody M2 agarose (Figure 5A). Gel filtration analysis showed that the purified proteins do not aggregate (Figure 5B). The purified proteins also maintained their strong PDL1-dependent CD40- and 41BB-agonism (Figure 5C).

The motivation to generate CD40- and 41BB antibody variants with PDL1-restricted agonism was not only to overcome the problematic FcγR-dependency of the agonism of conventional αCD40 and α41BB antibodies but also to link the agonism for these TNFRs directly with checkpoint blockade. As expected in view of the fact that the PDL1-specific Fab_2_ and scFv domains of the bispecific CD40/PDL1- and 41BB/PDL1 antibody variants were largely not affected in antigen binding (Table 1 and 2), the bispecific molecules efficiently inhibited binding of a soluble PD1-GpL fusion protein to PDL1-expressing cells (Figure 5D). Moreover, the constructs efficiently unleashed TCR-induced activation of a NFAT-regulated luciferase gene from the inhibitory effect of PD1 signaling in TCR^+^PD1^+^ Jurkat cells cocultivated with CHO-K1 cells expressing a membrane-bound TCR agonist and PDL1 (Figure 5E).

### α41BB-IgG1(N297A)-HC:scFvPDL1 enhances T-cell anti-tumor cell activity

**Figure 6:**
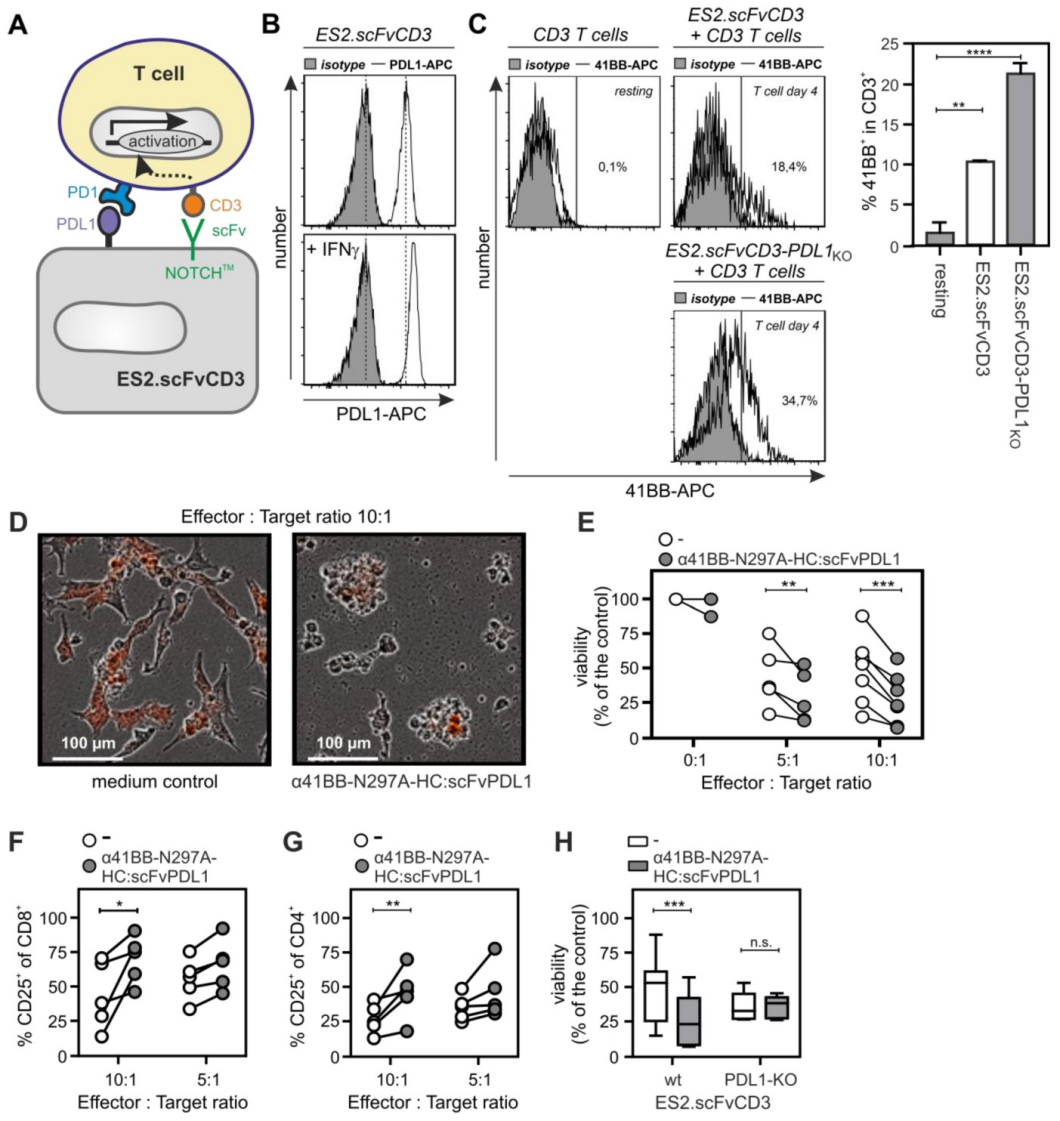
** T-cell costimulation by αCD3 expressing tumor cells and α41BB-IgG1(N297A)-HC:scFvPDL1. (A)** Scheme of experimental co-culture design.** (B)** FACS analysis of PDL1-expression on ES2.scFvCD3 ovarian cancer cells with and without IFNγ pretreatment. **(C)** Left panels: representative flow cytometry of 41BB cell surface expression on resting CD3^+^ T-cells, T-cells activated with ES2.scFvCD3 cells or activated with a PDL1-deficient variant of the latter. Right panel: Averaged results of experiments with 5 independent donors. **D-G** IFNγ-pretreated ES2-scFvCD3 cells were co-cultured with CD3^+^ T-cells in the indicated target to effector ratios with or without 10 µg/mL α41BB-IgG1(N297A)-HC:scFvPDL1. Microscopy was performed after 3 days **(D)**. Viability (**E**) and CD25 expression of CD4+ (**F**) and CD8+ cells (**G**) were analyzed on day 4. **(H)** ES2-scFvCD3 and the corresponding PDL1-KO variant were pretreated with IFNγ and co-cultured with CD3^+^ T-cells (E:T = 10:1) with or without 10 µg/mL α41BB-IgG1(N297A)-HC:scFvPDL1. After 4 days viability was analyzed. Shown are the results obtained with T-cells of 8 independent donors.

**Figure 7:**
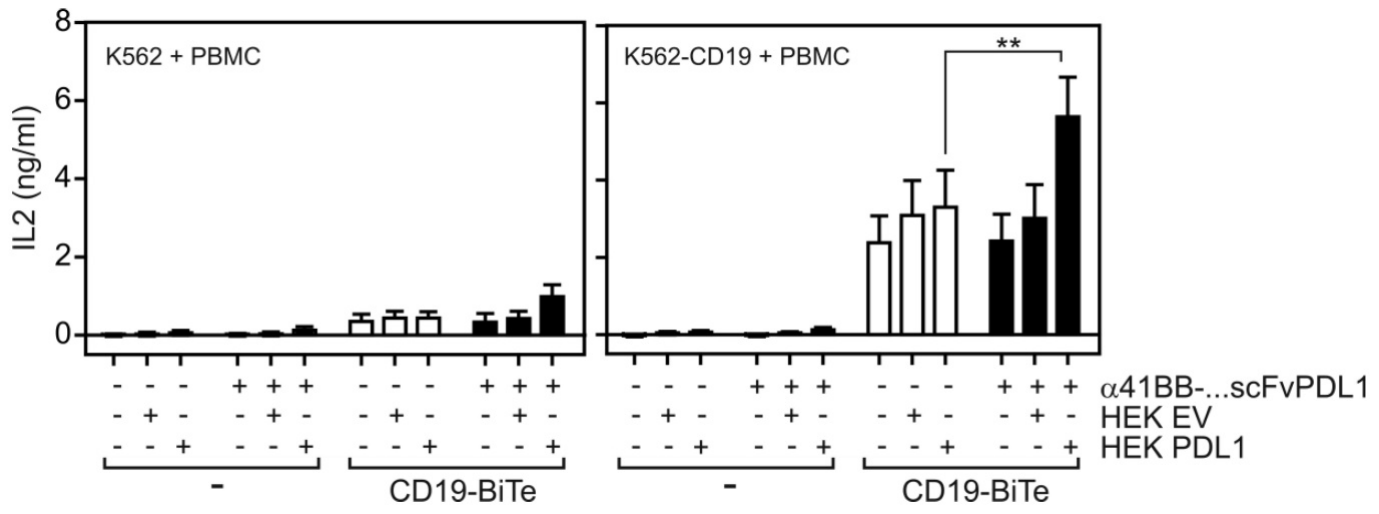
** T-cell costimulation by a CD19-BiTe and α41BB-IgG1(N297A)-HC:scFvPDL1.** K562 cells and K562-CD19 transfectants were seeded in 96-well plates (35 x 10^3^ cells/well). Next day, cells were challenged as indicated with PBMCs, Empty vector or PDL1 transfected HEK293 cells and α41BB-IgG1(N297A)-HC:scFvPDL1. After an additional day IL2 production was analyzed by ELISA. Shown are the averaged results of 6 independent experiments.

To evaluate the ability of α41BB-IgG1(N297A)-HC:scFvPDL1 to enhance the anti-tumor activity of T-cells, we took advantage of ES2-scFv:CD3 cells, a variant of the ovarian carcinoma cell line ES-2 stably expressing an agonistic plasma membrane-associated scFv:CD3-NOTCH-TM fusion protein (Figure 6A). PDL1 expression is high in IFNγ-treated ES2-scFv:CD3 cells and is further enhanced by treatment with IFNγ (Figure 6B). Cocultivation of PBMCs with ES2-scFv:CD3 cells for 3 days resulted in upregulation of 41BB in T-cells and the use of a PDL1-deficient variant of ES2-scFv:CD3 showed an even enhanced 41BB upregulation (Figure 6C). Treatment of IFNγ-supplemented PBMC/ES2-scFv:CD3 cocultures with α41BB-IgG1(N297A)-HC:scFvPDL1 revealed significant enhanced killing of ES2-scFv:CD3 cells which was not observed in cocultures with PDL1-deficient ES2-scFv:CD3 cells (Figure 6D-H). At a 10:1 effector : target cell ratio there was furthermore a significant increase in the frequency of CD25^+^ CD8^+^ and CD4^+^ T-cells (Figure 6F and 6G). In a second in vitro T-cell activation model, we treated cocultures of PBMCs, CD19-expressing K562 cells and HEK293-transfected PD-L1 cells with a low concentration of a CD19-specific BiTe resulting in CD19-restricted suboptimal T-cell activation (Figure 7). Again, cotreatment with α41BB-IgG1(N297A)-HC:scFvPDL1 enhanced the T-cell response in a PDL-dependent manner (Figure 7). In a variation of this experiment, we initially analyzed whether CD19 plus PDL1 coexpressing cells and a mixture of cells individually expressing CD19 and PDL1 perform differently in membrane PDL1-restricted enhancement of CD19-BiTe-induced T-cell activation by α41BB-IgG1(N297A)-HC:scFvPDL1. For this purpose, we analyzed α41BB-IgG1(N297A)-HC:scFvPDL1 costimulation of BiTe treated T-cells in cocultures with either empty vector transfected HEK293 cells plus CD19/PDL1 cotransfected HEK293 cells or CD19 transfected HEK293 cells plus PDL1 transfected HEK293 cells. In both scenarios there was again significantly enhanced IL2 production in the presence of α41BB-IgG1(N297A)-HC:scFvPDL1 (Figure S4). However, there was no significant difference between the usage of cells cotransfect with CD19 and PDL1 and mixtures of cells individually transfected with CD19 and PDL1 (Figure S4). This suggests that coexpression of CD19 and membrane PDL1 brings no additional benefit for α41BB-IgG1(N297A)-HC:scFvPDL1-induced costimulation of BiTe treated T-cells but to refined follow-up experiments are needed.

## Discussion

There is broad experimental evidence from various groups analyzing different bivalent αCD40 and α41BB antibodies that these antibodies regularly acquire strong agonistic activity upon binding to FcγRs but are typically not agonistic or even antagonistic in their “free” form 12,16-22. Experiments with signaling defective FcγR mutants, activating versus inhibitory FcγRs and FcγR non-immune cells suggest that the FcγR-dependent conditional mode of agonism of αCD40 and α41BB antibodies does not require FcγR signaling. This suggests that the sheer presentation in cell-bound form is the responsible factor for making αCD40- and α41BB antibodies agonistic. Of course, the fact that FcγR-bound αCD40 and α41BB antibodies act as TNFR agonists does not affect the initiation of FcγR signaling. Thus, the agonistic activity of conventional αCD40 and α41BB antibodies is in vivo typically superimposed by FcγR-associated activities.

In accordance with the hypotheses that plasma membrane-associated presentation is the major factor determining agonistic activity of conventional αCD40- and α41BB antibodies, we and others recently reported anchoring-dependent agonism of αCD40- and α41BB antibody fusion proteins carrying an anchor domain specific for plasma membrane-exposed tumor marker proteins, such as CD20 12, BCMA 13, FAP 23 or mesothelin 24. In this work, we exploit this mode of action to generate bifunctional αCD40- and α41BB antibody fusion proteins combining two synergistically acting activities, namely i) conditional FcγR-independent agonism and ii) PD1-PDL1 checkpoint inhibition. We genetically fused a PDL1 blocking scFv domain to the C-termini of the αCD40 antibody C and the α41BB antibody HBKK4 25 and observed strong membrane PDL1-conditioned CD40/41BB activation (Figure 2A and 3A) with full preservation of the checkpoint inhibitory activity of PDL1-scFv domain (Figure 5D and 5E). Noteworthy, not only αCD40- and α41BB antibody fusion proteins with a PD1 blocking scFv:PDL1 anchoring domain displayed strong dual activity but also αPDL1 antibody fusion proteins with C-terminal scFv:CD40 or scFv:41BB domains (Figure 2A, 3A, 5D and 5E).

Thus, it appears that the concrete molecular nature of the TNFR binding domain within a bispecific antibody fusion protein is of only secondary relevance. In line with this assumption, Hinner et al. 26 recently described strong anchoring dependent 41BB agonism with an αHer2-IgG4 fusion protein with a 41BB-specific anticalin binding domain at the C-terminus of the heavy chain and Mikkelsen et al. 27 observed strong anchoring dependent 41BB agonism with an CEA-41BB trimerbody. The conditional agonistic activity of plasma membrane-presented CD40- and 41BB-specific antibodies and antibody fusion proteins correspond well with the fact that the natural activators of CD40 and 41BB, CD40L and 41BBL, are membrane-bound molecules. Indeed, soluble 41BBL and CD40L molecules are poorly active but gain high conditional activity upon genetic fusion with an anchoring domain enabling plasma membrane-associated presentation mirroring the situation observed with αCD40- and α41BB antibodies 15,28-33. Thus, the molecular nature of a 41BB (or CD40) binding domain appears secondary for the construction of dual activity fusion proteins with conditional 41BB/CD40 agonism as long as the TNFR binding domain is linked with an anchoring domain enabling binding to the plasma membrane.

In sum, the reported findings confirmed the overwhelming relevance of plasma membrane attachment for the agonism of CD40- and 41BB-specific antibodies discussed above but also demonstrate a broadly practicable path for the generation of dual activity antibody fusion proteins combining CD40/41BB costimulation and checkpoint inhibition. Indeed, PDL1-targeted α41BB enhanced CD19-Bite induced T-cell activation (Figure 7) but also T-cell anti-tumor cell activity against genetically engineered tumor cells expressing membrane-bound αCD3 (Figure 6). Future in vivo studies must now show whether αCD40 and α41BB fusion proteins with a PDL1 blocking anchoring domain are superior with respect to anti-tumor efficacy and side effects compared to the combined application of PDL1 blocking antibodies and conventional CD40- and 41BB-specific antibodies triggering TNFR- and FcγR signaling.

## Conclusions

Taken together, we showed that per se non-agonistic antibody and antibody fragment fusion proteins bispecific for CD40 and PDL1 or 41BB and PDL1 acquire potent CD40 and 41BB agonism upon membrane PDL1 binding. This finding allowed the generation of fusion proteins linking PD1-PDL1 checkpoint blockade intimately with CD40 and 41BB activation. Since PDL1 expression is particular prominent in many tumors, the conditional membrane PDL1-restricted mode of CD40 and 41BB agonism furthermore promises particular efficient CD40/41BB activity in the tumor and thus reduced dose-limiting off-tumor immune stimulation. Further studies must now show whether such membrane PDL1-restricted CD40 and 41BB agonists are indeed superior to the co-application of systemically active CD40/41BB agonists and PDL1-blocking antibodies with respect to efficacy and side effects.

## Material and Methods

### Cell culture and reagents

HT1080 cells stably transfected with CD40 and 41BB 15, HEK293 cells (ATCC, Rockville, USA), ES-2 cells (ATCC, Rockville, USA), K562 and K562-CD19 were maintained in RPMI1640 medium (Sigma-Aldrich, Steinheim, Germany) complemented with 10% FCS (GIBCO, EU Approved, South America and Thermo Fischer). U2OS cells were cultivated in DMEM medium (Sigma-Aldrich, Steinheim, Germany) again supplemented with 10% FCS. Cell lines were grown at 37 °C and 5% CO_2_ and passaged every third or fourth day. Expression plasmids encoding 41BB and CD40 were a kind gift of Pascal Schneider (University of Lausanne). To obtain expression plasmids for membrane 41BBL and membrane CD40L the corresponding full-length DNA sequences were cloned into the pEYFP-C1 vector. The PDL1 encoding expression plasmid (pCMV6-XL4) was purchased from Origene Technologies, Inc. The scFv:CD3 presenting cell lines ES-2scFv:CD3 and its PDL1-deficient variant ES-2.PDL1KO.scFv:CD3 were generated using the Lentiviral synNotch receptor construct pHR_PGK_scFv:CD3_synNotch_Gal4VP64, which was obtained from the plasmid pHR_PGK_antiCD19_synNotch_Gal4VP64, a gift from Wendell Lim (Addgene plasmid # 79125;^34^), by replacing the antiCD19 scFv domain by a scFv of the antiCD3 antibody UCHT-1v9. Lentivirus was produced by transient transfection of HEK293T cells using the VSV-G packing system using FuGENE (Promega). Viral supernatants were collected, filtered through a 0.2-μm filter (Eppendorf) and used to transduce (1.5 mL supernatant) 2.5 × 10^5^ pre-seeded ES2 cells (6 well tissue culture plate (Corning), 1.5 mL) in the presence of 4 μg/mL polybrene (Sigma-Aldrich). Transduced cells were sorted for expression of the Myc-tag which precedes the extracellular scFv:CD3 domain using anti-Myc mAb Pacific Blue (clone 9B11, Cell Signaling) with a Sony cell sorter sh800s. ES-2scFv:CD3 cells stably expressing CD3 were also transduced with the Lentiviral vector pLKO.1 mCherry (Addgene plasmid # 128073) for visualization.

### Cloning and production of antibody fusion proteins

To construct expression plasmids encoding the heavy and light chains of the various Flag-tagged antibody variants, synthetic DNA fragments and PCR products were assembled in the pCDNA3.1 vector by standard cloning techniques (Table 3). Antibodies were produced by transient transfection of HEK293 cells with 1:1 mixtures of the expression plasmids encoding the heavy and light chain pair of interest using PEI (polyethylenimine; Polyscience Inc., Warrington, USA) as described elsewhere 35. To check the size and concentration of the various antibody fusion proteins, 15 µl supernatant were analyzed by western blotting using the αFLAG antibody M2 (Sigma-Aldrich, Saint Louis, USA) as primary antibody and goat αmouse-IgG1 IRDye 800CW antibody (Licor, Lincoln, USA). Antibody concentrations were obtained by comparison with a serial dilution of an in house FLAG-tagged control antibody of known concentration.

### Purification of antibody fusion proteins

The Flag-tagged antibody fusion proteins were purified from cell culture supernatants by affinity chromatography with anti-Flag M2 agarose (Sigma-Aldrich, Steinheim, Germany) as described in detail elsewhere for Flag-tagged scFv fusion proteins 36. In brief, after binding of the proteins to the anti-Flag M2 agarose gel, the columns were washed with TBS. To elute the bound Flag-tagged fusion proteins, the anti-Flag M2 agarose was washed with an excess of Flag® peptide. The eluted Flag-tagged proteins were dialyzed to exchange TBS against PBS and to reduce Flag® peptide concentrations. To control the purity of the antibody fusion proteins and to estimate their concentration, the purified fusion proteins and the Amersham's “Low Molecular Weight Calibration Kit for SDS Electrophoresis” (GE Healthcare UK Limited, Little Chalfont, UK) containing defined amounts of a-lactalbumin, trypsin inhibitor, carbonic anhydrase, ovalbumin, albumin and phosphorylase-b were separated by SDS-PAGE and visualized by silver staining (silver staining kit, Thermo Scientific, Rockford, USA). The comparison of the antibody band intensities with those of the standard proteins allowed estimation of antibody concentrations.

### Size Exclusion Chromatography

The purified antibody fusion proteins were analyzed by size exclusion chromatography (SEC) to check the aggregation and degradation. In general, the MAbPac™ SEC-1 HPLC column (Thermo Scientific, Rockford, USA) was pre-equilibrated with PBS at a flow rate of 0.76 mL/min. After the column pressure stayed stable, proteins (300 µL, 80-300 µg/mL) were manually injected into the injector and analyzed by UV at 280 nm. Calibration of the column was carried out with the column performance check standard aqueous SEC 1 solution (Phenomenex, Torrance, USA) containing bovine thyroglobulin (670 kDa), IgA (300 kDa), IgG (150 kDa), ovalbumin (44 kDa), and myoglobin (17 kDa).

### Coculture assays and IL8 ELISA

To evaluate the agonistic activity of the various αCD40- and α41BB antibody fusion proteins their ability to stimulate IL8 production, which crucially requires activation of the classical NFκB signaling pathway, was determined. For this purpose, cells that produce high amounts of IL8 in response to stimulation of CD40 and 41BB (HT1080-CD40 and HT1080-41BB transfectants, U2OS cells (endogenous CD40)) were seeded in 96-well plates (2 X 10^4^ cells / well). The next day, medium was changed and supplemented with a similar number of HEK293 cells transfected with empty vector or expression vectors encoding PD-L1 (or membrane CD40L/41BBL) along with the antibody fusion proteins of interest. The amount of IL8 in the supernatant, as an indicator of TNFR-mediated NFκB activation, was determined after overnight cultivation by help of an IL8 ELISA Kit (BD Biosciences, San Diego, USA). OD values were measured with a PHOmo photometer (anthos Mikrosysteme GmbH, Friesoythe, Germany).

### Equilibrium binding studies and heterologous competition assays

The affinity of the various antibody fusion proteins for CD40, 41BB and PD-L1 was determined by equilibrium binding studies with *Gaussia princeps* (GpL) luciferase tagged variants of these fusion proteins. HEK293 cells were transiently transfected with a CD40, 41BB or PD-L1 expression plasmid. Next day, cells were incubated with increasing concentrations of the various antibody fusion proteins to determine total binding. Empty vector (EV) transfected cells were treated in parallel in a similar fashion to measure unspecific binding. After incubation at 37 °C for 1.5 h, cells were washed three times with ice cold PBS, resuspended in 50 µl RPMI containing 0.5% FCS and transferred to a black 96-well plate to measure cell associated luciferase activities. 25 µl of the substrate coelenterazin (1.5 µM) (Carl Roth, Karlsruhe, Germany) diluted in PBS were added to each well and luminescence were directly measured with LUmo luminometer (anthos Mikrosysteme GmbH, Friesoythe, Germany). Finally, K_D_-values were calculated by subtracting the RLU values of the unspecific binding from total binding values to obtain the specific binding and the latter values were fitted by nonlinear regression using the “one side specific binding” function of the GraphPad Prism 5 software. Experiments with R^2^ values < 0.95 were excluded.

To analyze the blockade of the interaction of PDL1 with PD1 by constructs containing a Fab or scFv domain specific for PDL1, heterologous competition assays with PDL1-GpL and HEK293 cells transiently transfected with a PD1 expression plasmid were performed. PD1 transfectants were incubated for 1.5 h at 37 °C with a constant amount of PDL1-GpL mixed with increasing concentrations of the different PDL1-interacting antibody fusion proteins. Afterwards transfectants were washed three time with ice cold PBS and cell associated luciferase activity was measured as described above. The effectiveness of blocking of the PD1 interaction with PDL1-GpL (IC50 value) was calculated by nonlinear regression using the “log (inhibitor) vs response - variable slope” option of GraphPad Prism 5.

### PD1 inhibition assay

Inhibition of PDL1-induced PD1 signaling was evaluated with the PD-1/PD-L1 Blockade Bioassay from Promega (Madison, USA). The assay was performed according to the instructions of the manufacturer. Luminescence was detected with LUmo luminometer (anthos Mikrosysteme GmbH, Friesoythe, Germany). RLU values obtained were finally evaluated with the nonlinear regression “log (inhibitor) vs response - variable slope” function of GraphPad Prism 5.

### Flow cytometry

ES-2 and ES-2.PDL1KO cells were suspended in 100 µL of PBS, stained (30 min, 4 °C) with anti-PDL1-APC (Biolegend) or mouse IgG2a-APC (Biolegend) as isotype control, washed with PBS and analyzed by flow cytometry. After 4 days of coculture with ES-2scFv:CD3-mcherry cells, CD3^+^ T-cells were furthermore collected and stained for CD4 (anti-CD4-FITC; Immunotools), CD8 (anti-CD8-Pacific Blue; Beckman Coulter), CD25 (mouse anti-CD25-APC; Immunotools), mouse anti-41BB-APC (Immunotools) and with the zombie NIR fixable viability kit (Biolegend).

### Evaluation of T-cell costimulation by ES-2scFv:CD3-mcherry cells and 41BB-IgG1(N297A)-HC:scFvPDL1

Buffy coat blood samples were obtained from the Dutch Blood Bank Sanquin (agreement nr. NVT0465.01), diluted with PBS (1:2), layered on a Lymphoprep density gradient (Alere Technologies AS) and centrifuged according to the manufacturer's instructions. Peripheral blood mononuclear cells (PBMCs) were collected after centrifugation, washed with PBS, and resuspended in complete RPMI-1640. T-cells were isolated from PBMCs using an autoMACS Pro Separator (Miltenyi Biotec) using the panT Cell Isolation Kit (Miltenyi Biotec). After isolation, CD3^+^ T-cells were frozen at -80 °C in FCS containing 10% DMSO. The day of the assay, T-cells were thawed and resuspended in RPMI-1640 with 10% FCS. ES-2scFv:CD3-mcherry cells (1x10^3^ cells per well, 96 well plate) were incubated for 24 h with 10 ng/mL rh-IFNγ (Immunotools). Medium was then replaced by 200 µL of RPMI with 10% FCS containing CD3^+^ T-cells and the antibody construct of interest. Co-cultured CD3^+^ T-cells and mcherry^+^ cancer cells were imaged for mcherry fluorescence using the Incucyte S3 system (Essen BioScience) and analyzed using the Incucyte S3 software. Four pictures of each well were acquired and analyzed based on the Top-Hat segmentation method. Viability was calculated as mcherry area (µm² of mcherry/image) at the indicated time point normalized to time zero divided by the mCherry area of untreated ES-2scFv:CD3-mcherry cells.

### Evaluation of T-cell costimulation by anti-CD19 BiTE and 41BB-IgG1(N297A)-HC:scFvPDL1

Blood samples were obtained from the department of transfusion medicine at the University Hospital Wuerzburg. For isolation of the PBMCs, blood was mixed 1:1 with PBS and was slowly layered on top of 20 mL of Histopaque®-1077 Hybri-Max^TM^ (H8889; Sigma-Aldrich, Germany). PBMCs were separated from erythrocytes and plasma by centrifugation at 1200 g for 30 min without breaking. The PBMCs containing layer was transferred into a new falcon and was mixed with 50 mL of PBS. After centrifugation at 1200g for 5 min, the cell pellet was resuspended in 15 mL of Red Blood Cell Lysing Buffer Hybri-Max^TM^ (R7757; Sigma-Aldrich, Germany) and incubated for 15 min at RT. Cells were then centrifuged twice at 1200 g for 5 min. PBMCs were then resuspended in 10 mL RPMI1640 media supplemented with 10% FCS and divided to two cell culture flasks containing 70 mL RPMI1640 and 10% FCS. The next day, PBMCs (2.4x10^5^ per well) along with HEK293 cells (2x10^4^ cells/well) transiently transfected with empty vector or PDL1 were added to K562 and K562-CD19 cells (3.5 x 10^4^ cells/well, 96-well plate). The various cell cultures were stimulated with Blinatumomab (100 pM, Pharmacy of the University Hospital Würzburg) and anti-41BB-IgG1(N297A)-HC:scFv:PDL1 (1000 ng/mL) for 20 h at 37 °C and 5% CO_2_. Supernatants were finally analyzed for IL2 production as a readout of T-cell activation using the OptEIA^TM^ Human IL-2 ELISA kit from BD Biosciences (NJ, USA).

## Supplementary Material

Supplementary figures.

## Figures and Tables

**Table 1:** ** Binding affinities of investigated aCD40/PDL1-antibody fusion proteins.** For statistical analysis see supplemental data Figure S1.

Antibody Fusion Protein	Interaction	Number of Experiments	Single K_d_ (ng/mL)	Mean K_d_ (ng/mL)
αCD40-Fab_2_-GpL	CD40	3	830, 600, 780	740 ± 120
	PDL1	2	n.b.	-
αCD40-Fab_2_-HC:scFvPDL1-GpL	CD40	3	890, 600, 1260	920 ± 330
	PDL1	4	190, 90, 140, 250	170 ± 70
αCD40-N297A-GpL	CD40	3	1340, 430, 990	920 ± 460
	PDL1	2	n.b.	-
αCD40-N297A-HC:scFvPDL1-GpL	CD40	3	1490, 730, 1080	1100 ± 380
	PDL1	4	290, 290, 300, 240	280 ± 30
αPDL1-N297A-GpL	CD40	2	n.b.	-
	PDL1	6	220, 210, 130, 270, 300, 190	220 ± 60
αPDL1-N297A-HC:scFvCD40-GpL	CD40	3	1800, 1210, 1520	1510 ± 300
	PDL1	4	150, 80, 260, 270	190 ± 90

n.b.; no binding

**Table 2:** ** Binding affinities of different α41BB/PDL1-antibody fusion proteins.** For statistical analysis see supplemental data Figure S3.

Antibody Fusion Protein	Interaction	Number of Experiments	Single K_d_ (ng/mL)	Mean K_d_ (ng/mL)
α41BB-Fab_2_-GpL	41BB	5	2550, 1970, 1850, 980, 1710	1810 ± 560
	PDL1	3	n.b.	-
α41BB-Fab_2_-HC:scFvPDL1-GpL	41BB	5	1950, 2130, 1020, 2820, 2700	2120 ± 720
	PDL1	4	110, 200, 70, 140	130 ± 60
α41BB-N297A-GpL	41BB	5	390, 470, 210, 190, 480	350 ± 140
	PDL1	3	n.b.	-
α41BB-N297A-HC:scFvPDL1-GpL	41BB	5	910, 790, 380, 590, 480	630 ± 220
	PDL1	4	240, 260, 200, 330	260 ± 50
αPDL1-N297A-GpL	41BB	2	n.b.	-
	PDL1	6	220, 210, 130, 270, 300, 190	220 ± 60
αPDL1-N297A-HC:scFv41BB-GpL	41BB	4	400, 770, 180, 280	410 ± 260
	PDL1	4	330, 390, 150, 370	310 ± 110

n.b.; no binding

**Table 3:** Domain architecture of heavy and light chain constructs used for the expression of antibody variants used in this study.

Construct	Architecture
α41BB-LC	S^a^ - L^1^ - F^b^ - L^3^ - V_L_(WO2006/126835 A1, aa sequence HBBK4-75L) - L^4^ - C_L_^c^
α41BB-LC-GpL	S^a^ - L^1^ - F^b^ - L^3^ - V_L_(WO2006/126835 A1, aa sequence HBBK4-75L) - L^4^ - C_L_^c^ - L^5^ - G^e^
α41BB-HC:IgG1(N297A)	S^a^ - L^1^ - F^b^ - L^2^ - V_H_ (WO2006/126835 A1, aa sequence HBBK4-75G1) - L^6^ - C_H_^d^
α41BB-HC:IgG1(N297A)-scFv:PD1L	S^a^ - L^1^ - F^b^ - L^2^ - V_H_ (WO2006/126835 A1, aa sequence HBBK4-75G1) - L^6^ - C_H_^d^ - L^5^ - VH(PDB 5GRJ_H) - L^7^ - L^8^ - L^1^ - VL(PDB 5GRJ_L)
α41BB-HC:FAB_2_	S^a^ - L^1^ - F^b^ - L^2^ - V_H_ (WO2006/126835 A1, aa sequence HBBK4-75G1) - L^6^ - C_H1_^f^
α41BB-HC:FAB_2_-scFv:PD1L	S^a^ - L^1^ - F^b^ - L^2^ - V_H_ (WO2006/126835 A1, aa sequence HBBK4-75G1) - L^6^ - C_H1_^f^ - L^5^ - VH(PDB 5GRJ_H) - L^7^ - L^8^ - L^1^ - VL(PDB 5GRJ_L)
αCD40-LC	S^a^ - L^1^ - F^b^ - L^3^ - V_L_ (US2016222124A1, see seq ID:36) - L^4^ - C_L_^c^
αCD40-LC-GpL	S^a^ - L^1^ - F^b^ - L^3^ - V_L_ (US2016222124A1, see seq ID:36) - L^4^ - C_L_^c^ - L^5^ - G^e^
αCD40-HC:IgG1(N297A)	S^a^ - L^1^ - F^b^ - L^2^ - V_H_ (US2016222124A1, see seq ID:37) - L^6^ - C_H_^d^
αCD40-HC:IgG1(N297A)-scFv:PD1L	S^a^ - L^1^ - F^b^ - L^2^ - V_H_ (US2016222124A1, see seq ID:37) - L^6^ - C_H_^d^ - L^5^ - VH(PDB 5GRJ_H) - L^7^ - L^8^ - L^1^ - VL(PDB 5GRJ_L)
αCD40-HC:FAB_2_	S^a^ - L^1^ - F^b^ - L^2^ - V_H_ (US2016222124A1, see seq ID:37) - L^6^ - C_H1_^f^
αCD40-HC:FAB_2_-scFv:PD1L	S^a^ - L^1^ - F^b^ - L^2^ - V_H_ (US2016222124A1, seq ID:37) - L^6^ - C_H1_^f^ - L^5^ - VH(PDB 5GRJ_H) - L^7^ - L^8^ - L^1^ - VL(PDB 5GRJ_L)
αPD1L-LC	S^a^ - L^1^ - F^b^ - L^3^ - V_L_ (PDB 5GRJ_L) - L^4^ - C_L_^c^
αPD1L-LC-GpL	S^a^ - L^1^ - F^b^ - L^3^ - V_L_ (PDB 5GRJ_L) - L^4^ - C_L_^c^ - L^5^- G^f^
αPD1L-HC:IgG1(N297A)	S^a^ - L^1^ - F^b^ - L^2^ - V_H_ (PDB 5GRJ_H) - L^6^ - C_H_^d^
αPD1L-HC:IgG1(N297A)-scFv:41BB	S^a^ - L^1^ - F^b^ - L^2^ - V_H_ (PDB 5GRJ_H) - L^6^ - C_H_^d^ - L^5^ - VH (WO2006/126835 A1, aa sequence HBBK4-75G1) - L^7^ - L^8^ - L^1^ - VL (WO2006/126835 A1, aa sequence HBBK4-75L)
αPD1L-HC:IgG1(N297A)-scFv:CD40	S^a^ - L^1^ - F^b^ - L^2^ - V_H_ (PDB 5GRJ_H) - L^6^ - C_H_^d^ - L^5^ - VH (US2016222124A1, see seq ID:37) - L^7^ - L^8^ - L^1^ - VL (US2016222124A1, see seq ID:36)

^1^ L = Linker: QL, for cloning purposes encoded by Mfe1 (CAATTG) ^2^ L = Linker: EF, for cloning purposes encoded by EcoR1 (GAATTC) ^3^ L = Linker: EL ^4^ L = Linker: GS, for cloning purposes encoded by BamH1 (GGATCC) ^5^ L = Linker: LE, for cloning purposes encoded by Xho1 (CTCGAG) ^6^ L = Linker: RS ^7^ L = Linker: RS, for cloning purposes encoded by Bgl2 (AGATCT) ^8^ L = Linker: STKGPKLEEGEFSEA ^a^ S = Signalpeptide: MNFGFSLIFLVLVLKGVQCEVKLVPR ^b^ F = Flag tag: DYKDDDDK ^c^ C_L_ = constant light chain (aa: 105-214, GenBank: BAA97671.1) ^d^ C_H_ = constant heavy chain of human IgG1 (aa:145-476, GenBank AAA02914.1) with A to N mutation at position 327 ^e^ G = *Gaussia princeps* luciferase (aa: 18-185, GenBank AAG54095) ^f^ C_H1_ = constant heavy chain of human IgG1 (aa: 145-260, GenBank AAA02914.1)

## Abbreviations

41BBL: 4-1BB Ligand
ADCC: antibody dependent cell cytotoxicity
Bite: Bi-specific T-cell engagers
CD40: cluster of differentiation 40
CD40L: CD40 Ligand
CDC: Complement dependent cytotoxicity
EV: Empty vector
Fab: Fragment antigen binding
FcγR: Fc gamma receptor
GpL: *Gaussia princeps* luciferase
IgG1: Immunoglobulin 1
IL8: Interleukin-8
scFv: Single chain variable fragment
PBMCs: Peripheral blood mononuclear cells
PD1: Programmed death 1
PDL1: PD1 Ligand 1
TNF: Tumor necrosis factor
TNFRSF: TNF receptor superfamily
TNFR: TNFRSF receptor
