# Supplementary material for: CD40- and 41BB-specific antibody fusion proteins with PDL1 blockade-restricted agonism
Source: Theranostics. 2022 Jan 1;12(4):1486–99. doi: 10.7150/thno.66119 (PMC8825603; doi:10.7150/thno.66119)
Supplement: Supplementary file 1 — Supplementary figures. [file thnov12p1486s1.pdf]

Supplemental data: Medler et al., CD40- and 41BB-specific antibody fusion proteins with PDL1 blockade-restricted agonism

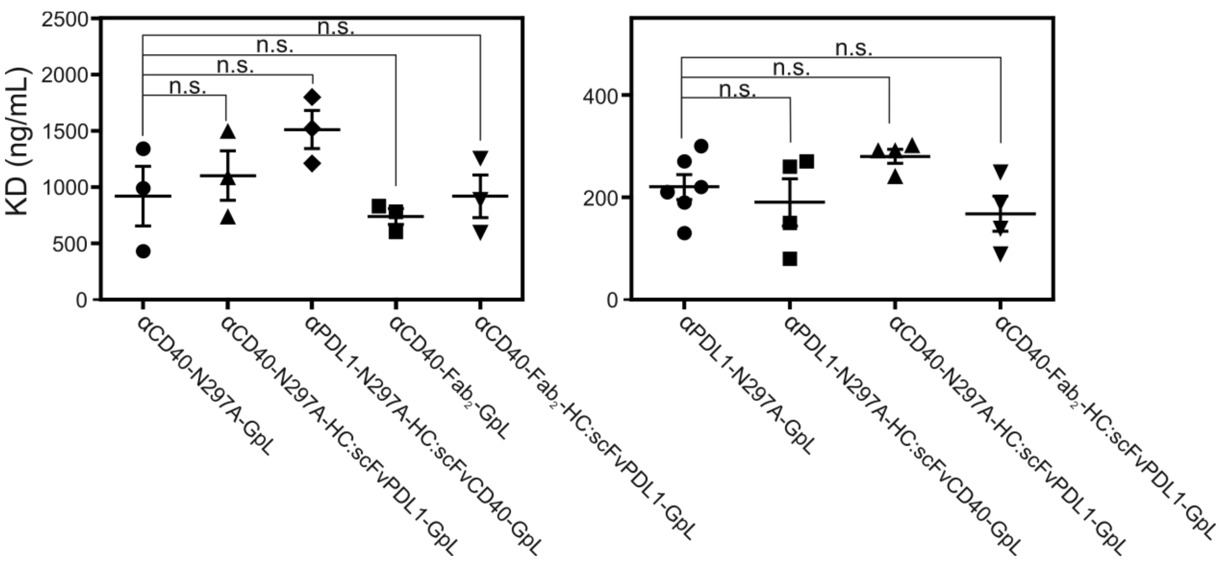

Figure S1. The Affinities of the CD40- and PDL1-interacting binding domains of CD40/PDL1-bispecific constructs are not affected by their domain architecture. The K_D-values listed in Table 1 for the various CD40/PDL1-bispecific constructs and the αCD40-Fab$_2$ molecule were compared with those of the parental antibody variants αCD40-IgG1(N297A) and α41BB-IgG1(N297A) according to Bonferroni's test. n.s., not significant.

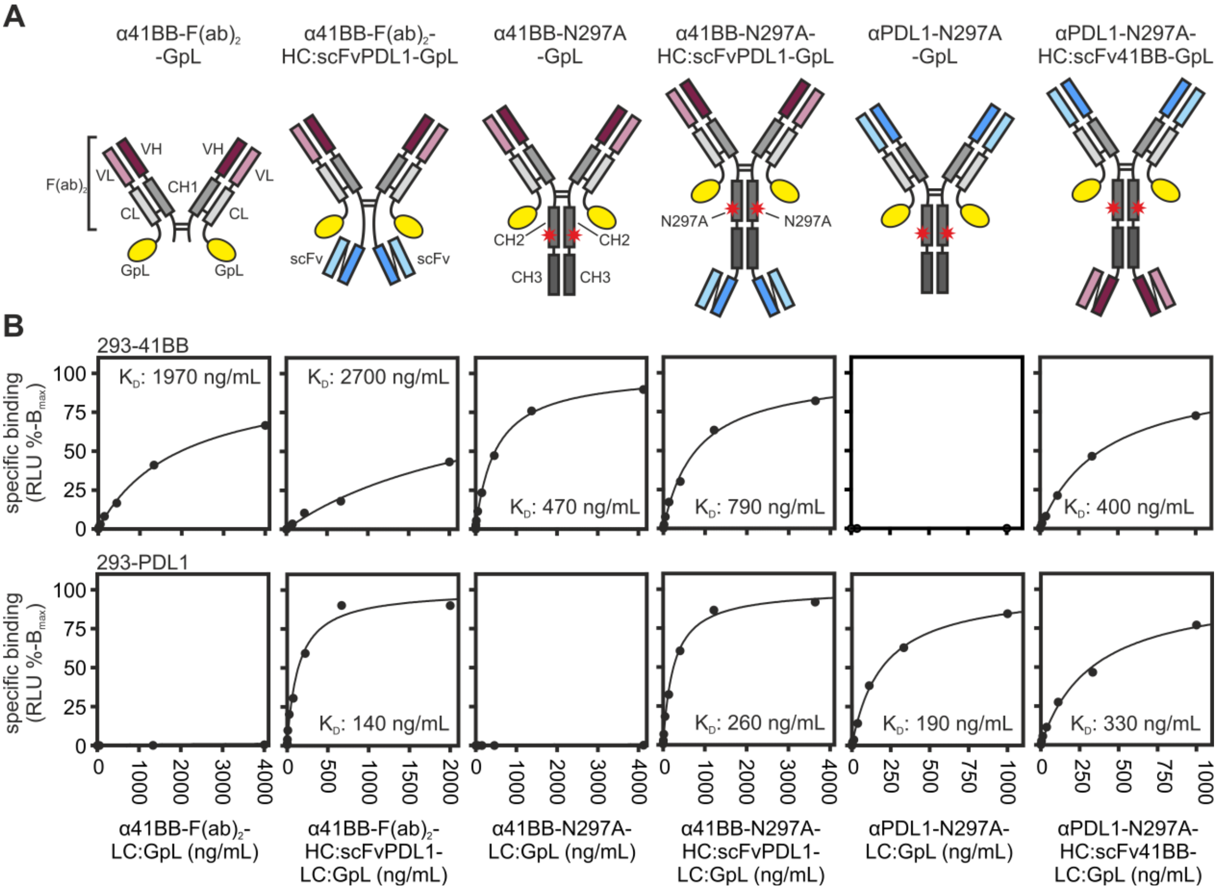

Figure S2. Equilibrium binding of 41BB/PDL1-bispecific antibody variants and the corresponding parental antibodies to 41BB and PDL1. (A) Domain architecture of the GpL-antibody fusion proteins used. **(B)** Specific binding of the constructs shown in A to HEK293 cells transiently expressing 41BB and PDL1. One representative experiment is shown. Mean and single K_D-values of four to six independent experiments are summarized in table 2 in of the manuscript.

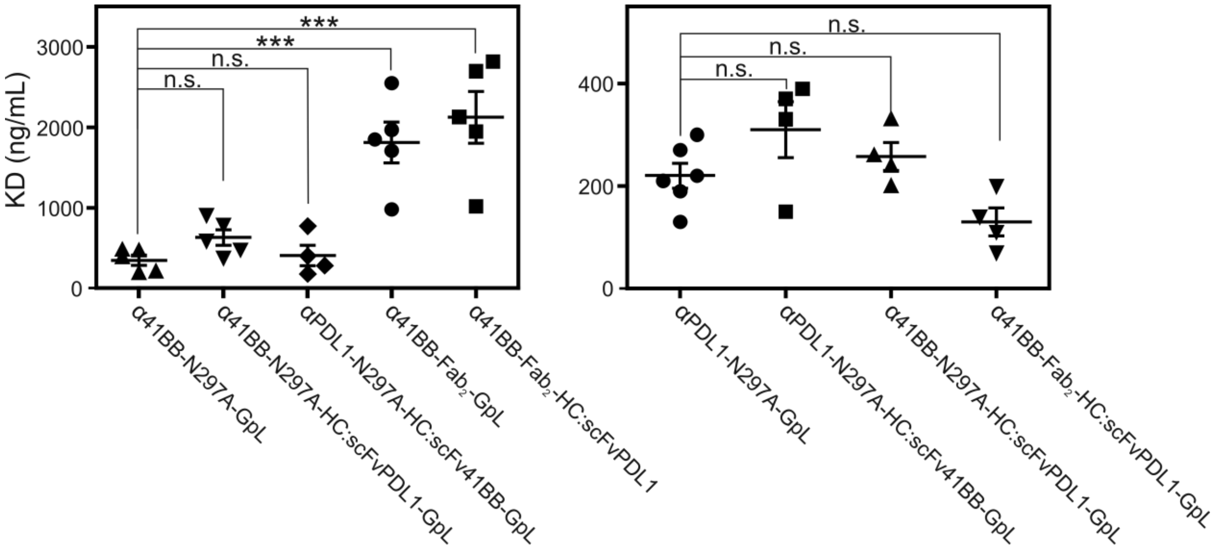

Figure S3. Affinities of the various 41BB/PDL1-bispecific antibody fusion proteins and α41BB-Fab$_2$ for 41BB and PDL1. The K_D-values listed in Table 2 for the various 41BB/PDL1-bispecific constructs and the α41BB-Fab$_2$ molecule were compared with those of the parental antibody variants α41BB-IgG1(N297A) and α41BB-IgG1(N297A) according to Bonferroni's test. Please note, the Fab$_2$ format for the 41BB binding domain correlates with a moderately but significantly reduced affinity. *** $p < 0.001$; n.s., not significant.

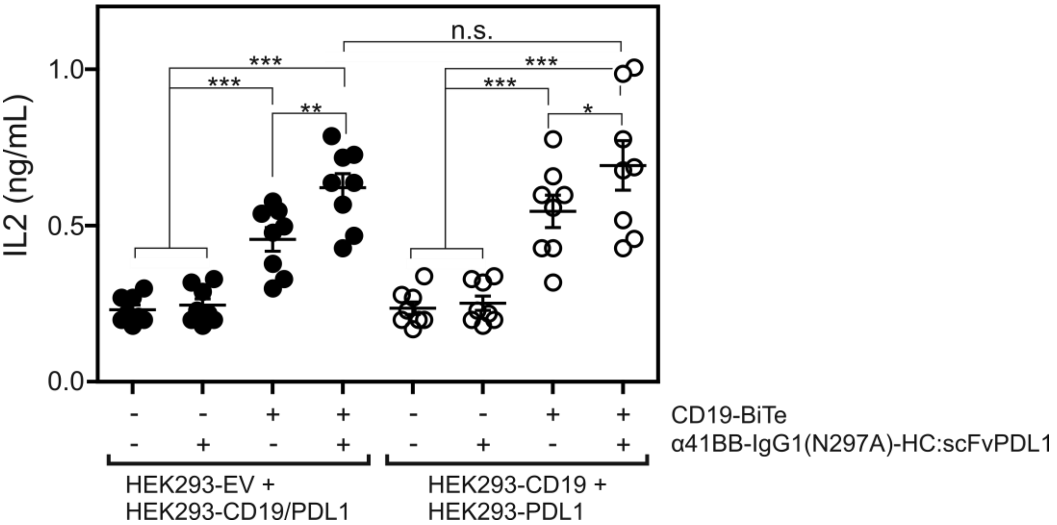

Figure S4. T-cell costimulation by a CD19-BiTe and α41BB-IgG1(N297A)-HC:scFvPDL1 in cocultures with cells coexpressing CD19 and PDL1 and cells expressing CD19 and PDL1 separately. 1:1 mixtures of HEK293 cells transfected with empty vector (EV) and CD19 plus PDL1 (filled circles) and 1:1 mixtures of HEK293 cells transfected with CD19 and PDL1 (open symbols) were seeded in 96-well plates (4 x 10^4 cells/well). Next day, cells were incubated with PBMCs (24x10^4 cells/well), 5 ng/mL CD19-BiTe and 100 ng/mL α41BB-IgG1(N297A)-HC:scFvPDL1 as indicated. After an additional day, IL2 production was analyzed by ELISA. Shown are the averaged results obtained with 7 independent donors. *** $p < 0.001$;** $p < 0.01$; * $p < 0.05$; n.s., not significant.
